# Gold nanoparticles/over-oxidized poly-eriochrome black T film modified electrode for determination of arsenic

**DOI:** 10.55730/1300-0527.3508

**Published:** 2022-10-08

**Authors:** Mürşide Ceren AFŞAR, Aydan ELÇİ, Zekerya DURSUN

**Affiliations:** Department of Chemistry, Faculty of Science, Ege University, Bornova, İzmir, Turkey

**Keywords:** Over-oxidized poly-eriochrome black T, gold particles, arsenic, modified electrode, electrode characterized

## Abstract

A glassy carbon electrode, modified with gold nanoparticles/over-oxidized poly-eriochrome black T film, was fabricated for sensitive determination of As(III) by stripping voltammetry in an acidic medium. The electrode surface properties were characterized by cyclic voltammetry, electrochemical impedance spectroscopy, scanning electron microscope (SEM), and X-ray photoelectron spectroscopy (XPS). The electrode demonstrated a good response towards As(III), with a detection of 0.077 *μ*M and good linearity in the range of 0.1 to 10 μM (R^2^ = 0.9977). For the determination of As(III), the fabricated electrode was placed in different mineral water samples, acidified with 0.75 M HCl solution, spiked with various concentrations of As(III). Spiked recoveries for mineral water samples were obtained in the range of 100.3%–105.0%. The relative standard deviations were lower than 4.0%.

## 1. Introduction

Arsenic is a constituent part of the Earth’s crust and is widely distributed across the environment, in the air, water, and land. While it exists in both inorganic and organic forms, it is more toxic in its inorganic form [1]. Arsenic contamination (as inorganic and organic species) in the groundwater, is a serious global threat to human health. Among various arsenic species, the inorganic species, arsenite (As(III)) and arsenate (As(V)) are predominantly found in water sources. In various samples, arsenite is present in larger quantities than arsenate [2]. While the oxide compounds of arsenic cause different types of cancer, arsenite has about 10–20 times higher toxic effects than arsenate species [3–5]. Although the maximum amount of arsenic allowed in drinking water is 10 μg L^−1^ as per the World Health Organization, the amount can vary from 7 to 50 μg L^−1^ in different countries [6]. Due to the high variation of concentration, accurate and precise detection of arsenic in drinking water samples has become a major interest of analytical chemists.

The amount of arsenic is usually detected by hydride generation atomic fluorescence spectrometry [7], hydride generation atomic absorption spectrometry [8], hydride generation inductively-coupled plasma mass spectrometry [9], high-performance liquid chromatography with chemical vapor generation [10], and electrospray MS [11]. On the other hand, chemical analysis systems are costly to use and sustain, and require fully equipped laboratories and complicated sampling processes. In addition, the electrochemical techniques seem to be a good alternative for arsenic determination due to various beneficial features, including high sensitivity, rapid analysis, ease of use, and low-cost instrumentation [12–16]. For electrochemical determination of arsenic in real samples, various types of electrodes (mercury drop, platinum, and gold) were applied by anodic [14,16,17] or cathodic stripping voltammetry [18]. Among the electrodes, gold is the most convenient electrode material for arsenic detection due to its high hydrogen overpotential and favorable reversibility. In particular, suitable applications of carbon-based electrodes incorporated with gold nanoparticles (AuNPs) have been described in several recent papers [13,16,17]. In addition, polymer films obtained by modifying the electrodes with simple and fast electrochemical polymerization techniques have garnered immense interest due to several features, such as higher physical stability, reproducibility, powerful adhesion to the electrode surface and improved electrocatalytic activity. Poly(solochrome black T) [19] and poly-eriochrome black T (pEBT) [20–23] as polymeric films, have been used for the analysis of multiple drugs, including dopamine, acetaminophen, acyclovir, epinephrine, adenine, and guanine. It is well known that the pEBT film on the surface of glassy carbon electrode (GCE) efficiently decreases the charge transfer resistance of the electrode and improves the electron transfer kinetics between the analytes and the electrodes [24]. On the other hand, it is known that the AuNPs change the oxidation potential of analytes and increase the oxidation current, and accordingly, the selectivity and sensitivity are improved [25]. So, it has been concluded that the synergistic interaction of pEBT and AuNPs can be advantageous for the electrochemical analysis. However, the applications of AuNPs-modified pEBT/GCE (AuNPs/pEBT/GCE) are limited for various drugs analysis [24–26].

In the present study, we have introduced an easy voltammetric method for arsenic determination using an AuNPs-modified pEBT_ox_/GCE.

Various analytical measuring systems were used to identify the chemical, morphological, and electrical properties of the AuNPs-modified pEBT_ox_/GCE by X-ray photoelectron spectroscopy (XPS), cyclic voltammetry (CV), electrochemical impedance spectroscopy (EIS), and field emission scanning electron microscopy (FE-SEM). The interaction of pEBT_ox_ and AuNPs, as well as glassy carbon, caused an increase in the electron transfer rate and the current signal. The determination of arsenic by AuNPs/pEBT_ox_/GCE provided a low detection limit, good selectivity, and sensitivity. The procedure was performed to detect arsenic in different mineral water samples. To the best of our knowledge, this is the first report about AuNPs-modified pEBT_ox_/GCE to detect As(III).

## 2. Materials and methods

### 2.1. Materials

Analytical grade Eriochrome black T, HCl (37%), HClO_4_ (70%), HAuCl_4_, and ethanol (99%) were purchased from Merck or Sigma-Aldrich. NaOH (pellets, purity ≥ 98%) was supplied from Riedel. All solutions were prepared using ultra-pure water (Milli-Q 18.2 MΩ cm, Millipore System Inc.). Highly pure nitrogen was used to remove oxygen from the solution.

### 2.2. Instruments and characterization

All electrochemical measurements were performed with a three-electrode system in Autolab PGSTAT 302N Electrochemical Analyser. GCE (3 mm diameter and 0.0707 cm^2^ geometric area) poly-Eriochrome black T (pEBT), over-oxidized poly-Eriochrome black T (pEBT_ox_), gold nanoparticles/poly-Eriochrome black T (AuNPs/pEBT), and gold nanoparticles/over-oxidized poly-Eriochrome black T (AuNPs/pEBT_ox_) modified GCE were used as the working electrodes, Ag/AgCl (sat. KCl) as the reference electrode, and platinum wire as the counter electrode. The morphologies and compositions of the electrode surfaces were identified using field emission scanning electron microscopy (FE-SEM; Gemini500, Carl Zeiss, Germany) and X-ray photoelectron spectroscopy (Thermo K–Alpha–Monochromated high-performance XPS spectrometer).

### 2.3. Preparation of over-oxidized poly(EBT) film covered GCE

Initially, the bare GCE was polished in the range of 3.0–0.05 microns Al_2_O_3_ slurry on a synthetic cloth. The electrode was then rinsed with distilled water and sonicated for 3 min in a mixture of ethanol and ultra-pure water (1:1 v/v). The electrochemical polymerization of Eriochrome black T monomer on the GCE surface (pEBT/GCE), was performed with CV over 20 repetitive potential cycles in the potential range of 0.5 V to 1.50 V (scan rate: 0.1 V s^−1^) from 1.0 mM Eriochrome black T solution. To improve the surface properties, an over-oxidation procedure at +0.6 V for 100 s was applied to the pEBT/GCE in 0.1 M NaOH solution. Finally, the Au nanoparticles were formed on the pEBT_ox_/GCE surface by CV in 0.10 M HCl and 3.0 mM HAuCl_4_ solution with repetitive potential cycling in the range of −0.8 V to 0.6 V accompanied by 0.1 V s^−1^scan rate over about 15 cycles.

### 2.4. Sample analysis

Mineral water samples from three different companies were obtained from the local stores. The method accuracy was controlled by detection of arsenic in mineral water samples.

## 3. Results and discussion

### 3.1. Electrode preparation and characterization

The electrochemical polymerization of EBT was shown in Figure S1a. During the electrochemical polymerization, two irreversible oxidation peaks were observed at 0.06 V and 0.66 V for EBT without the corresponding cathodic processes in the first reverse current-potential scan. A decrease in the peak height was observed during the oxidation of EBT, with the sequential current-potential cycles, accompanied by the polymerization occurring on the electrode surface [27–29]. Although the pEBT film containing −COOH, −OH, −SO_3_^−^groups were formed on the GCE surface, the pEBT/GCE surface did not exhibit enhanced surface porosity and electroconductivity. To improve the surface properties, the pEBT film on the GCE surface was over-oxidized at +0.6 V in 0.1 M NaOH solution (Figure S1b) [30–34].

On the other hand, it is known that the synergic interaction of pEBT and AuNPs increases the electron transfer rate and current signal, which resulted in high sensitivity and selectivity [26,35]. The surface of pEBT_ox_/GCE was modified with AuNPs for higher sensitivity and selectivity. The voltammograms obtained with AuNPs/pEBT_ox_/GCE are indicated in Figure S1c.

A cathodic peak was observed at −0.40 V, related to the Au^3+^ ion reduction. A small oxidation peak appeared at −0.15 V, corresponding to the oxidation of the Au metal particles to Au^+^, in addition to the main oxidation peak at 0.18 V, representing the conversion of Au metal particles to Au^3+^. These peaks indicate that the AuNPs were deposited on the pEBT_ox_/GCE successfully. The obtained electrode was denoted as AuNPs/pEBT_ox_/GCE.

Figure S2 shows the voltammetric behaviour of the bare GC, the pEBT/GC, the pEBT_ox_/GC, the AuNPs/pEBT/GC and the AuNPs/pEBT_ox_/GC electrodes in 0.1 M KCl solution containing 5.0 mM K_3_[Fe(CN)_6_]/K_4_[Fe(CN)_6_]. Reversible redox behaviour was observed for all the electrodes. The best redox behaviour was observed for AuNPs/pEBT_ox_/GCE, owing to the enhanced surface properties with the over-oxidation process [31] and Au particle-deposition. To demonstrate the same, the active surface areas of AuNPs-pEBT_ox_/GCE, pEBT_ox_/GCE and bare GCE were obtained using the Randles–Sevcik equation, as 0.574, 0.063 and 0.041 cm^2^, respectively. Obviously, after the modification with the gold nanoparticles and over-oxidation of pEBT on the bare GCE, the most active and the highest surface area were obtained. This result could be explained by the interaction of arsenic with the gold nanoparticles on AuNPs/pEBT_ox_/GCE [12,23,24,26].

**Figure 1:**
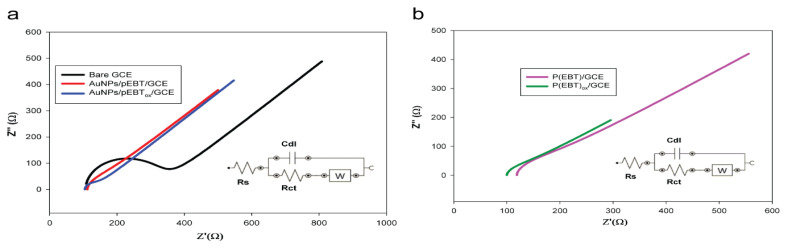
Electrochemical impedance spectroscopy curves (EIS) of a. the bare GCE (black line), AuNPs/pEBT/GCE (red line), AuNPs/pEBT_ox_/GCE (blue line), b. pEBT/GCE (pink line) and pEBT_ox_ (green line) in the presence of 5.0 mM Fe(CN)_6_^3−^/Fe(CN)_6_^4−^ (1:1) + 0.1 M KCl. Inset: Equivalent electrical circuit.

EIS studies revealed reliable data regarding the interfacial electrical properties of the electrode surfaces. Figure 1 indicates that the EIS behavior of all the electrodes in the presence of 0.1 M KCl solution contains 5.0 mM K_3_[Fe(CN)_6_]/K_4_[Fe(CN)_6_] at varying frequencies, ranging from 0.10 to 50,000 Hz. The typical Nyquist diagram of equivalent circuits involving bare GC, pEBT/GC, pEBTox/GC, AuNPs/pEBT/GC and AuNPs/pEBTox/GC electrodes are depicted in Figure 1, including ohmic resistance (Rs), Warburg impedance (W), double layer capacitance (Cdl) and electron transfer resistance (Rct) of the electrolyte solution. The EIS data obtained was matched with this equivalent circuit to extract the values of the Rct and other components of the EIS measurement (Table S1). For the bare GCE (Figure 1a), a large semicircle domain with the charge transfer resistance (Rct) of 217 Ω in the Fe[CN]_6_^3−/4−^ redox probe, was observed. On the other hand, the Rct values of the pEBT/GC, pEBT_ox_/GC, AuNPs/pEBT/GC, and AuNPs/pEBT_ox_/GC electrodes (Figure 1a and b) decreased clearly to 62.8 Ω, 37.5 Ω, 50 Ω, and 33.9 Ω, respectively. The deposition of Au particles on pEBT_ox_/GCE exhibited a lower Rct value, indicating the synergistic effect of Au particles and over-oxidized pEBT film towards the redox process of the Fe[CN]_6_^3−/4−^ redox probe.

**Figure 2:**
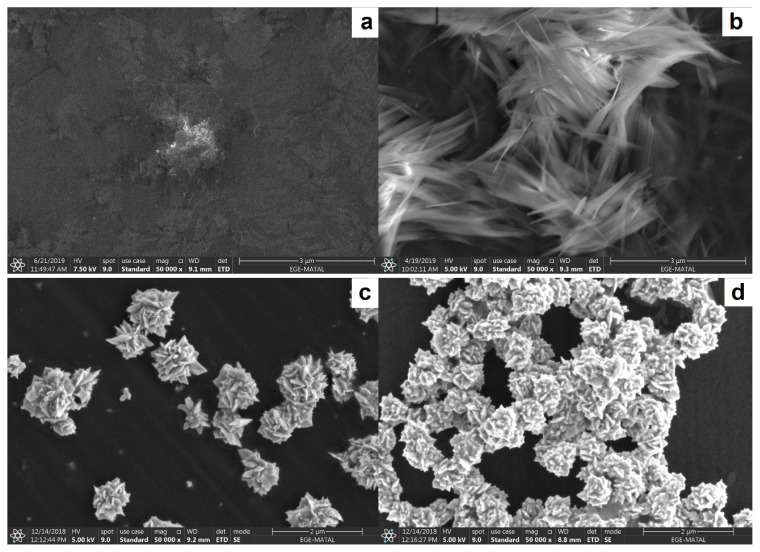
Scanning electron microscope (SEM) images of a. pEBT/GCE, b. pEBT_ox_/GCE, c. AuNPs/pEBT/GCE, d. AuNPs/pEBT_ox_/GCE.

The morphology and porosity of the pEBT, pEBT_ox_, AuNPs/pEBT, AuNPs/pEBT_ox_ films were further clarified by SEM. Figure 2a shows that the pEBT film is very smooth and homogeneous. However, for the pEBT_ox_ film (Figure 2b), the surface porosity clearly improved by the generation of functional carboxyl groups (−COO/−COOH). AuNPs/pEBT film surface is different as light dots are noticeable and pEBT film seems transparent. Au/NPs dimensions were in the range of 100–200 nm (Figure 2c). The SEM image of the AuNPs/pEBT_ox_ film indicates that the nanoparticle size (75–80 nm) was smaller due to the over-oxidation of the polymer film (Figure 2d).

XPS studies were performed to identify the chemical composition of pEBT, pEBT_ox,_ AuNPs/pEBT, and AuNPs/pEBT_ox_ modified GCE surfaces.

**Figure 3:**
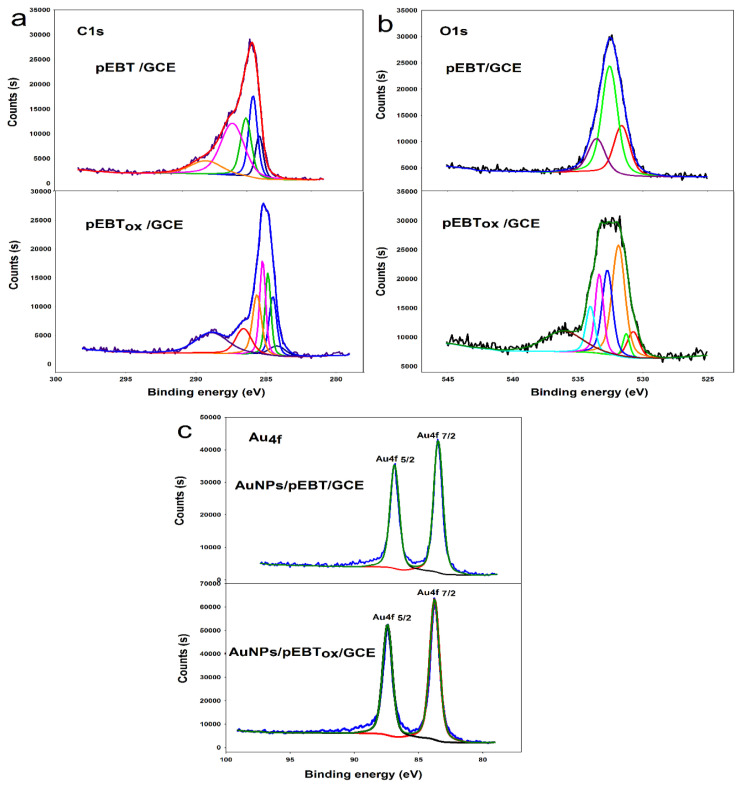
X-ray photoelectron spectroscopy (XPS) high-resolution spectra of a. C1s, b. O1s, c. Au4f of pEBT/GCE and pEBT_ox_/GCE.

The C(1s) spectrum of pEBT/GCE was evaluated at 284.057–284.5 eV (sp^2^ hybridised carbon), 285.179 eV (sp^3^ hybridised carbon), 286.242 eV (−C-O), and 288.173 eV (−O-C=O) respectively [31,36]. The considerable diversity in the form of C(1s) spectrum of pEBT_ox_/GCE was observed at 284.63 and 284.912 eV (sp^2^ hybridised carbon), 285.33–285.75 eV (sp^3^ hybridised carbon), 286.445 eV (−C-O) and 289.1 eV (−O-C=O). As expected, C1s peak belonging to the −COOH/COO^−^ functional groups was significantly higher as compared to pEBT (Figure 3a).

The O1s signal of the pEBT/GCE and pEBT_ox_/GCE are represented in Figure 3b. The O1s spectrums of pEBT/GCE and pEBT_ox_/GCE are depicted in Figure 3b. The curve fits of the O1s peaks for pEBT and pEBT_ox_ observed at 531.5, 532.5, and 533.5 eV, and 530.9, 531.8, 532.7, 533.2, 534.0, and 536.7 eV, respectively (Figure 3b). The peaks located at 530.9 eV, 531.5 eV, and 531.8 eV can be attributed to the −C=O bond in the polymer chain while the far peaks located at 532.7 eV and 532.5 eV corresponded to the −O-C bond in the polymeric rings. Other oxygen species at 533.5, 533.2, and 534.0 eV could be attributed to the COOH/COO^−^ groups on the surface. Substantial differences between the shapes and the location of the peaks can be observed for the O(1s) signals of pEBT/GCE and pEBT_ox_/GCE. A new noticeable peak appeared at 536.2 eV after over-oxidation of pEBT. The new signal could be ascribed to the COOH/COO^−^ functionalities in pEBT_ox_ [37,38].

Figure 3c shows the Au 4f core level XPS spectra of the AuNPs/pEBT and AuNPs/pEBT_ox_ modified GCE surfaces. The spectra show two main peaks at 87.60 and 84.00 eV for AuNPs/pEBT and 87.40 and 83.88 eV for AuNPs/pEBT_ox_ corresponding to the Au 4f_5/2_ and Au 4f_7/2_ orbits, respectively. The peaks corresponded to the metallic Au. The binding energy shifts were obtained at 0.2 eV for Au 4f_5/2_ and 0.12 eV for Au 4f_7/2_ compared with the bulk Au metal due to the interaction difference between the Au metal and pEBT/pEBT_ox_ [39,40].

### 3.2. Voltammetric behaviour of arsenic (III) at bare and modified electrodes

**Figure 4:**
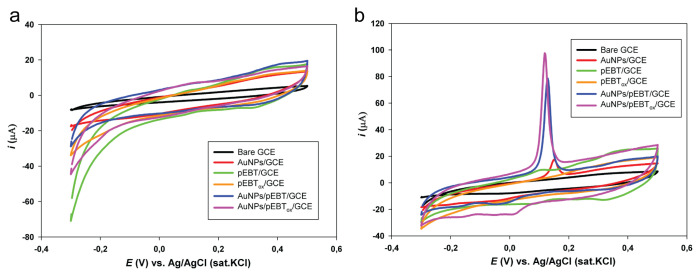
The cyclic voltammetric (CV) behaviour of all the electrodes in 0.75 M HCl solution in a. absence, b. presence of 0.1 mM As(III) with a scan rate of 0.05 V s^−1^ for the bare GC, AuNPs/GC, pEBT/GC, pEBT_ox_/GC, AuNPs/pEBT/GC, AuNPs/pEBT_ox_/GC electrodes.

The voltammetric behaviour of bare GC, pEBT/GC, pEBT_ox_/GC, AuNPs/pEBT/GC and AuNPs/pEBT_ox_/GC electrodes were studied in the absence and presence of 0.1 mM As(III) in HCl solution (0.75 M) by CV with a scan rate of 0.05 V s^−1^ (Figure 4). In the absence of As(III), there was no oxidation peak in the working potential range for the bare GCE and the modified GCEs (Figure 4a). In the presence of 0.1 mM As(III) and 0.75 M HCl solution, a well-defined irreversible oxidation peak was formed at 0.128 V, accompanied with the 71.12 μA peak current and 0.118 V with 85.28 μA peak current, and 0.148 V with 12.87 μA peak current on AuNPs/pEBT/GC, AuNPs/pEBT_ox_/GC and AuNPs/GC electrodes, respectively (Figure 4b). The peak currents for the AuNPs/pEBT/GCE and AuNPs/pEBT_ox_/GCE significantly increased by about 5.52 times and 6.62 times for As(III) as compared to AuNPs/GCE (0.148 V with the peak current of 12.87 μA). The improvement in the oxidation current was ascribed to the increase in the electroactive surface area (A_act_) by the Au modification which enhanced the sensitivity of the modified electrode. On the other hand, the bare GC electrode showed no ox/red peaks in 0.1 mM As(III) and 0.75 M HCl solution whereas the pEBT/GC and pEBT_ox_/GC electrodes exhibited two small characteristic peaks for oxidation/reduction at ~0.10 V and 0.35 V, respectively.

From the significantly improved signal of arsenic oxidation at the modified electrodes, it can be concluded that the electrodes containing gold NPs are the most suitable because offavorablee As-Au interactions [41].

### 3.3. Optimization of AuNPs/pEBT_ox_/GCE surface

To find the best experimental conditions for the DPV determination of As(III), several basic parameters incorporated in the fabrication of the over-oxidized pEBT_ox_ film, AuNPs, and solution properties were also estimated. The parameters included the EBT monomer concentration, EBT polymerization cycle number, electrochemical over-oxidation potential and the time of pEBT and the number of cycles for the Au deposition. Optimum parameters, described by estimating the oxidation current value of arsenic, were detected as follows: 1.0 mM EBT in 0.1 M HClO_4_, 20 cycles for electropolymerization of EBT, 0.1 M NaOH concentration, 100 s over-oxidation time, 0.6 V over-oxidation potential, and 15 cycles for Au deposition from 3.0 mM HAuCl_4_ solution on pEBT_ox_/GCE in 0.75 M HCl, respectively.

### 3.4. Optimization of supporting electrolyte concentration

For the detection of arsenic with DPV, the influence of HCl concentration on the oxidation current was examined for AuNPs/pEBT_ox_/GCE. The change in the peak current of 0.1 M As(III) by varying HCl concentrations (Figure S3) showed that a higher peak current was obtained in 0.75 M HCl. Thus, 0.75 M HCl was preferred as the supporting electrolyte for further studies.

### 3.5. The scan rate effect on voltammetric behaviour of AuNPs/PEBTox/GCE in the presence of As(III)

Scan rate experiments demonstrated significant information about the electrode processes. Therefore, AuNPs/pEBT_ox_/GCE was operated at the scan rates of 0.01 to 0.50 V s^−1^ in the presence of 1.0 μM As(III) (Figure S4). As indicated in Figure S4a, the oxidation current (I) increased linearly proportional to the square root of the scan rate, as per the linear regression equation, I (μ Α) = 5.2805 v^1/2^ (V s^−1^) – 4.2884 (R^2^ = 0.997). This data demonstrates that the voltammetric behavior of the AuNPs/pEBT_ox_/GCE reactions in the presence of As(III) was controlled by diffusion. On the other hand, the linear relationship between E_pa_ and ln ν was described by E_pa_ = 0.0177 lnν +0.0633 with a correlation coefficient R^2^ equal to 0.981, as shown in Figure S4b. Using the slope of Figure S3a and Laviron equation [42]:


(1) $$E_{pq}=E^{0}+m\;\left[0.78+\text{ln}(D^{\frac{1}{2}}\;k_{s}^{-1}-0.5\;\text{ln }m)\right]+\frac{m}{2}\;\text{ln }v$$

where


(2) $$m=[\frac{RT}{(1-\alpha )n\alpha \text{F}}]$$

electron transfer coefficient value was obtained as 0.522 that confirms an irreversible process for electro-oxidation of As(III).

### 3.6. Optimization of differential pulse voltammetric measurement parameters

In the DPV studies, the influence of the accumulation potential, time, pulse amplitude, and scan rate on the voltammetric behavior of 5.0 μM As(III) in 0.75 M HCl were evaluated. The effect of the accumulation potential (E_acc_) on the oxidation current of 5.0 μM As(III) was studied in the potential range of −0.10 to −0.35 V vs. Ag/AgCl with 250 s of deposition on AuNPs/pEBT_ox_/GCE (Figure S5a) in 0.75 M HCl solution. The results in the Figure S5a demonstrate that the highest oxidation current was obtained at −0.3 V which suddenly dropped at more negative potential (−0.35 V) due to the hydrogen gas evolution on AuNPs/pEBT_ox_/GCE. Thus, −0.3 V was considered as the accumulation potential for the following experiments.

The influence of the accumulation time (t_acc_) on 5.0 μM As(III) was studied from 30 to 500 s under the optimum conditions. As can be seen in Figure S5b, the arsenic stripping oxidation current increased on increasing the accumulation time to 250 s, and subsequently remained constant up to approximately 350 s. The surface saturation occurred on the electrode after 250 s. As a result, 250 s was distinguished as the optimum accumulation time for the further studies.

The influence of the potential amplitude (E_amp_) on the stripping oxidation current of As(III) was examined from 0.01 V to 0.125 V in Figure S5c. The optimum amplitude value was found to be 0.075 V. The effect of various scan rates was studied in the range of 0.002 and 0.04 V s^−1^ (Figure S5d). The best oxidation current was obtained at 0.01 V s^−1^ scan rate.

### 3.7. Linear range and detection limit

**Figure 5:**
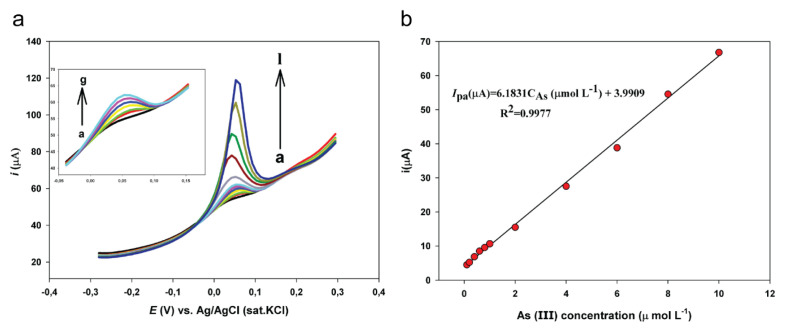
a. DP stripping voltammograms for various concentrations of As(III) in 0.75 M HCl at AuNPs/pEBT_ox_/GCE with 0.01 V s^−1^ scan rate; As(III) concentrations: a. blank, b. 0.10 μM, c. 0.20 μM, d. 0.40 μM, e. 0.60 μM, f. 0.80 μM, g. 1.0 μM, h. 2.0 μM, i. 4.0 μM, j. 6.0 μM, k. 8.0 μM, l. 10.0 μM, b. Calibration curve of As(III) for AuNPs/pEBT_ox_/GCE.

Under the optimized conditions, As(III) was detected on the AuNPs/pEBT_ox_/GCE using DPV. Figure 5a indicates the DPV response of arsenic in the concentration range of 0.10 μM to 10.0 μM based on the linear regression equation (Figure 5b):


(3) $$I_{pa}\;(\mu A)=6.1831C_{As}\;(\mu \text{M})+3.9909\;({\text{R}}^{2}=0.9977)$$

The limit of detection (LOD) was calculated as 0.0775 μM by the equation, LOD = 3.3 σ/m, (σ: the standard deviation of the response for the blank solution, m: the slope of the calibration curve). Additionally, DPV studies were performed for As(III) on AuNPs/pEBT/GC and Au disc electrodes. The peak current increased linearly with the increase in As(III) concentration from 0.4 μM to 10.0 μM (Figure S6) as per the linear regression equation:


(4) $$I_{pa}\;(\mu A)=3.17776C_{As}\;(\mu \text{M})+7.179\;({\text{R}}^{2}=0.9911\;\text{and }LOD=1.179\;\mu \text{M})$$

for AuNPs/pEBT/GCE, and 1.0 μM to 10.0 μM with linear regression equations of:


(5) $$I_{pa}\;(\mu A)=1.5781C_{As}\;(\mu \text{M})+3.4530\;({\text{R}}^{2}=0.99601\;\text{and LOD}=0.702\;\mu \text{M})$$

for Au disc electrodes, respectively. These results indicate that AuNPs/pEBT_ox_/GCE shows higher activity and sensitivity for As(III) and an improved detection limit compared with both AuNPs/pEBT/GCE and Au disc electrodes.

### 3.8. Reproducibility, repeatability, and stability of AuNPs/pEBT_ox_/GCE

The sensitivity, reproducibility, repeatability, and stability of the modified electrodes are important parameters for their applications with real samples. Under the same experimental conditions, a minimum of five different electrodes were fabricated to test the reproducibility of the preparation of AuNPs/pEBT_ox_/GCE electrode. Intra-day (n = 5) and inter-day (n = 5) measurements were carried out for 1.0 μM As(III) in 0.75 M HCl solution. The relative standard deviations (RSDs) for intra-day and inter-day measurements were calculated as 2.80% and 1.34%, respectively. The repeatability of the composite electrode was also examined for 1.0 μM As(III) in 0.75 M HCl solution for five consecutive measurements. The RSD value was calculated as 4.07%. Based on the obtained RSDs, it is concluded that AuNPs/pEBT_ox_/GCE has good reproducibility. The peak current for 1.0 μM As(III) on AuNPs/pEBT_ox_/GCE was measured for five days and 92.10% of the original peak current was maintained for consecutive five days. Consequently, the modified electrode can be used for at least five days without any remarkable error.

### 3.9. Interference study

In order to test the selectivity of AuNPs/pEBT_ox_/GCE, the DPV behavior of 1.0 μM As(III) was evaluated for potential interference coexisting metal ions (Table 1). A metal ion was considered to be an interferent when it caused an error greater than ±10% in the analytical signal of As(III). The results are given in Table 1.

The experimental results indicate that 100-fold amounts of Na^+^ and K^+^ and one-fold amounts of Cd(II), Ni(II), and Hg(II) have no remarkable change on the signal of arsenic. However, the highest positive interference was obtained for arsenic anodic stripping peak current in the presence of more than one-fold amount of Sb(III) ion. Cu(II) has caused a major interference in the determination of arsenic. A 10-fold excess of Cu(II) caused a decrease in the peak current of As(III). The oxidation peak at about +0.20 V, can be ascribed to the intermetallic compound oxidation that occurred during the accumulation of arsenic in the presence of Cu(II) as Cu_x_As_y_ [42].

### 3.10. Analytical applications

AuNPs/pEBT_ox_/GCE was utilized in the mineral water samples to determine arsenic with the standard addition method. Since the natural arsenic concentration in the samples was below the LOD value of the AuNPs/pEBT_ox_/GC electrodes, recovery studies were performed to prove the accuracy by spiking the mineral water samples with As(III) in the concentration range of 0.199–5.960 **μM** (Table 2). The recovery values varied from 100.3% to 105%. The obtained recovery results demonstrate that the matrix of the mineral water samples does not interfere with the arsenic current signal under these experimental conditions.

The analytical performance of AuNPs/pEBT_ox_/GCE and the published data are summarised in Table 3. The electrode demonstrated better sensitivity and detection limit as compared to the listed studies.

## 4. Conclusion

Gold nanoparticles-modified pEBT_ox_/GCE was fabricated via electrochemical synthesis. The chemical, morphological, and electrical features of the modified electrode surface, were defined by XPS, SEM, and EIS and confirmed the presence of the modifiers on the AuNPs/pEBT_ox_/GCE surface. The modified electrode exhibited better electrical conductivity and a higher active surface area in comparison with AuNPs/pEBT/GCE and bare GCE, as confirmed by the CV and EIS measurements. DPV studies for the determination of As(III) on AuNPs/pEBTox/GCE, demonstrated that the optimum analytical performance of the electrode constitutes low detection limit, good selectivity, and reproducibility. Although there were some more sensitive published papers than the present study, the study indicated that easily and quickly fabricated and long storage stability of AuNPs/pEBT_ox_ GC electrode can be used for reliable, cost-effective, and selective differential pulse anodic stripping analysis of As(III) ion in mineral water samples. Another advantage is that quite good recovery studies performed with spiked samples were in the range of 99.5%–105.2% compared with the published papers [49].

## Data availability statement

The data that support the findings of this study are available from the corresponding author upon reasonable reques

## Supplementary Information

Figure S1 a. Cyclic voltammograms of EBT polymerization on GCE in 0.1 mol L^−1^ HClO_4_ containing 1.0 mmol L^−1^ EBT from 1st cycle to 20^th^ cycle. Inset: 1^st^ scan, b. Overoxidation of poly(EBT) at 0.6 V on GCE in 0.1 mol L^−1^ NaOH solution, c. Cyclic voltammograms of Au NPs deposition at pEBT_ox_/GCE in a 0.1 mol L^−1^ HCl solution. Scan rate: 0.1 mV s^−1^.

Figure S2 Voltammetric behaviour of the bare GC, the pEBT/GC, the pEBT_ox_/GC, the AuNPs/pEBT/GC and the AuNPs/pEBT_ox_/GC electrodes in 0.1 M KNO_3_ solution containing 5.0 mM K_3_[Fe(CN)_6_]/K_4_[Fe(CN)_6_].

Figure S3 Cylic voltammograms for electooxidation of 0.1 mmol L^−1^ As(III) on electrode at various HCl concentrations.

Figure S4 a. The plots of oxidation peak currents of As(III) versus the square root of scan rate, b. The relationship between peak potential and ln ν.

Figure S5 Effect of a. Deposition potential, b. deposition time, c. pulse amplitude, d. scan rate on peak currents of 5.0 μmol L^−1^ As(III) in 0.75 mol L^−1^ HCl.

Figure S6 Calibration curve of As(III) for AuNPs/pEBT/GC and Au disk electrodes.

**Table S1:** Data obtained after fitting the electrochemical impedance spectra with the equivalent circuit

	R(Ω)	C(μF)	Rct (Ω)	W(μMho)
Bare GCE	107	1.47	217	819
pEBT/GCE	119	35.2	62.8	958
pEBT _ox_/GCE	99.8	29.5	37.5	950
AuNPs/pEBT/GCE	112	39.8	50.0	943
AuNPs/pEBT _ox_/GCE	105	6.23	33.9	961

## Figures and Tables

**Table 1:** Effect of coexisting ions in 1.0 μM As(III) Analysis (n = 3).

Molar ratio of arsenic to interferent/Recovery %				
Coexisting ions	1:1	1:10	1:100	1:1000
Na^+^	100.51	90.12	91.07	80.26
K^+^	99.13	96.79	95.45	92.26
Hg^2+^	99.73	34.53	-	-
Cu^2+^	78.29	13.26	-	-
Sb^3+^	84,17	83,32	-	-
Cd^2+^	99.35	88.82	78.81	63.46
Ni^2+^	94.99	74.21	57.40	46.58

**Table 2:** Determination of As(III) on AuNPs/pEBT_ox_/GCE in spiked mineral water samples (n = 3).

Samples	As(III) Added (μM)	As(III) Found (μM)	Recovery %	RSD %

Mineral water (Aegean Region)	n.d.	n.d.	-	-
	0.199	0.200	100.5	0.74
	0.396	0.411	103.7	1.33
	0.998	1.040	104.2	2.13
	5.960	5.980	100.3	0.55

Mineral water (Marmara Region)	n.d.	n.d.	-	-
	0.199	0.198	99.5	2.21
	0.396	0.399	100.8	1.33
	0.998	1.050	105.2	0.48
	5.960	6.010	100.8	0.74

Mineral water (Central Anatolia Region)	n.d.	n.d.	-	-
	0.199	0.202	101.5	1.30
	0.396	0.398	100.5	0.76
	0.998	1.010	101.2	1.87
	5.960	6.060	101.7	0.89

n.d.: not detected

**Table 3:** Comparison of AuNPs/pEBT_ox_/GCE for As(III) determination with other modified electrodes with similar nanocomposites.

Electrodes	Supporting electrolyte	Detection limits μg L^−1^	RSD %	Voltammetric detection type	Linear range	Reference
Nano gold-crystal violet film GCE	phosphate buffer (pH 7)	59.93	≤0.4	Anodic stripping	2.0–22.0 μM	[14]
Chitosan-Fe(OH)_3_ modified GCE	0.10 M acetate buffer (pH 5.2)	5.4	5.87	Differential pulse anodic stripping	2–100 ppb	[43]
Nano-TiO_2_ modified gold strip electrode	3.0 M HCl	10	3.3	Linear sweep	10–80 mg L^−1^	[44]
Graphene oxide decorated gold microelectrode	pH 6.0 PBS solution (0.10M)	12.15	2.12	Square wave anodic stripping	1–10 ppb	[45]
Carbon paste modified with carbon nanotubes (CNTPE) and polymeric resins.	50% (v/v) ethanolic supporting electrolyte, 0.4 mol L^−1^ HClO_4_	10.3	5	Linear sweep and differential-pulse voltammetry	30 to 110 mg L^−1^	[46]
Exfoliated graphite Bismuth electrode.	0.1 M KNO_3_ (pH 6)	5	5	Square wave anodic stripping	20–100 mg L^−1^	[47]
Stable iridium-modified boron-doped diamond electrode	0.1 M phosphate buffer solution (pH 3)	347.61	≤0.4	Cyclic	1–100 **μM**	[48]
AuNPs/EBT_ox_/GCE	0.75 M HCl	5.80	≤4.1	Anodic stripping	0.10 to 10.0 μM	This work
