# Supplementary figures and images for: Gold nanoparticles/over-oxidized poly-eriochrome black T film modified electrode for determination of arsenic
Source: Turk J Chem. 2022 Oct 8;46(6):2123–34. doi: 10.55730/1300-0527.3508 (PMC10446921; doi:10.55730/1300-0527.3508)

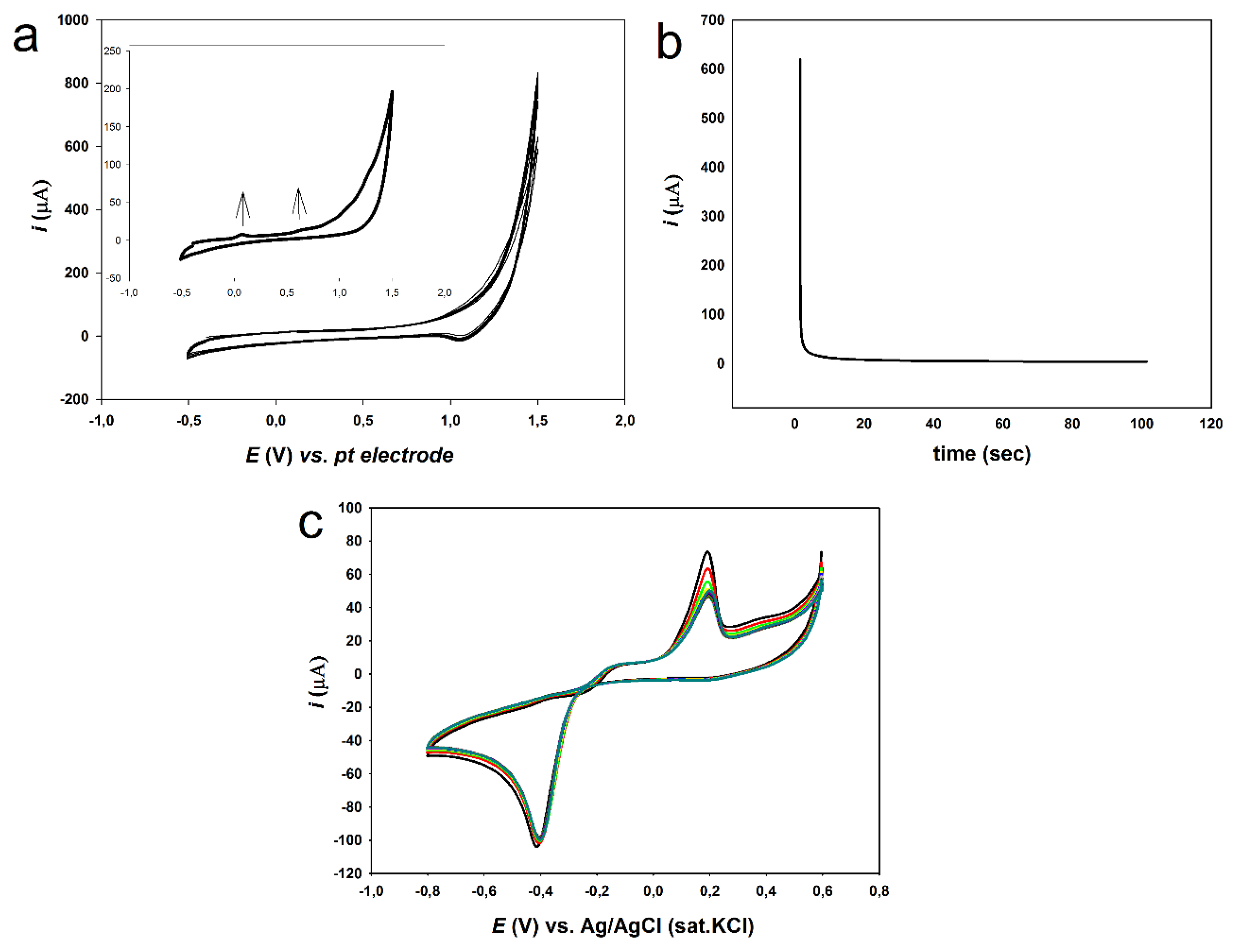

Supplement: Figure S1 — a. Cyclic voltammograms of EBT polymerization on GCE in 0.1 mol L−1 HClO4 containing 1.0 mmol L−1 EBT from 1st cycle to 20th cycle. Inset: 1st scan, b. Overoxidation of poly(EBT) at 0.6 V on GCE in 0.1 mol L−1 NaOH solution, c. Cyclic voltammograms of Au NPs deposition at pEBTox/GCE in a 0.1 mol L−1 HCl solution. Scan rate: 0.1 mV s−1. [file turkjchem-46-6-2123s1.tif]

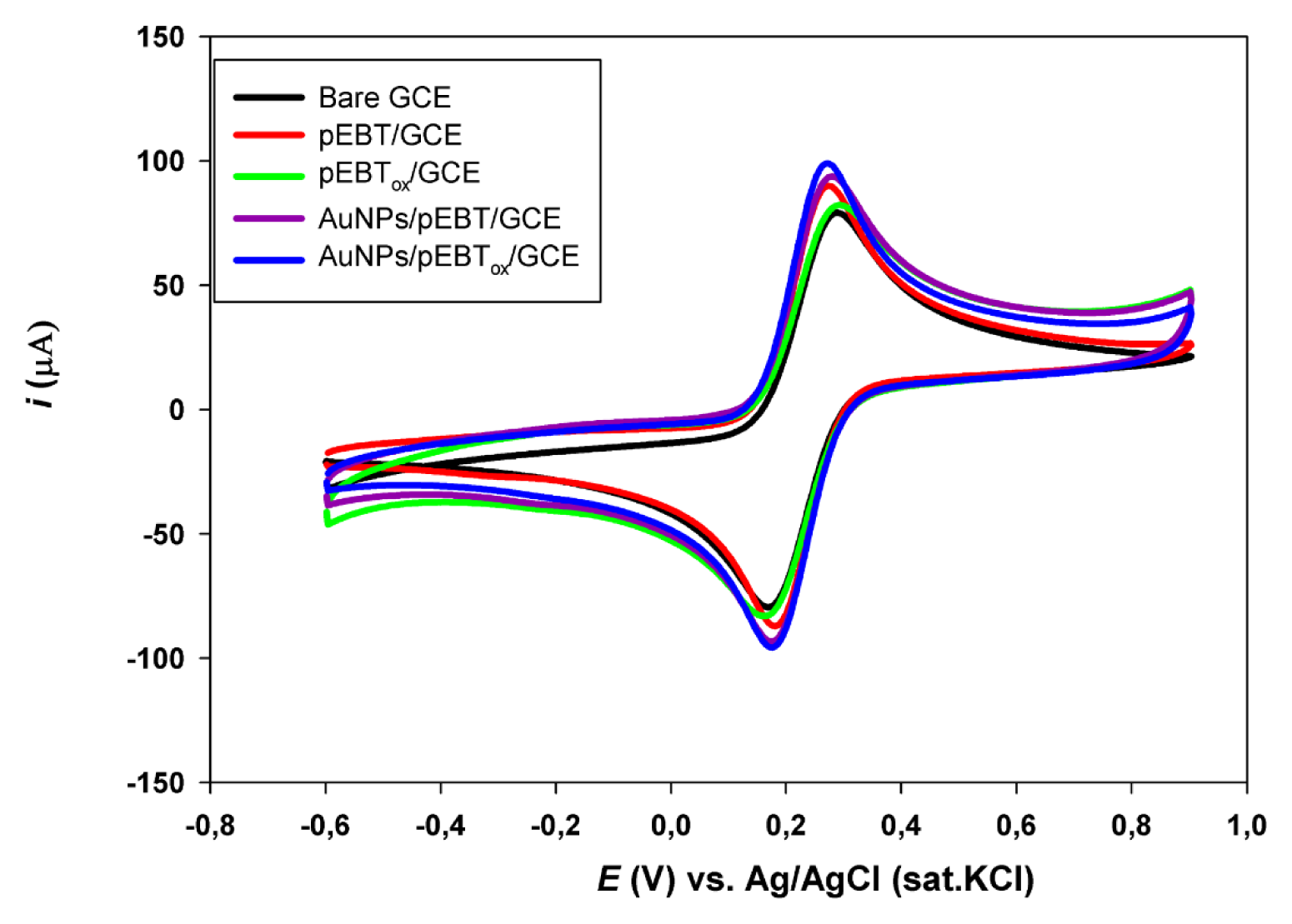

Supplement: Figure S2 — Voltammetric behaviour of the bare GC, the pEBT/GC, the pEBTox/GC, the AuNPs/pEBT/GC and the AuNPs/pEBTox/GC electrodes in 0.1 M KNO3 solution containing 5.0 mM K3[Fe(CN)6]/K4[Fe(CN)6]. [file turkjchem-46-6-2123s2.tif]

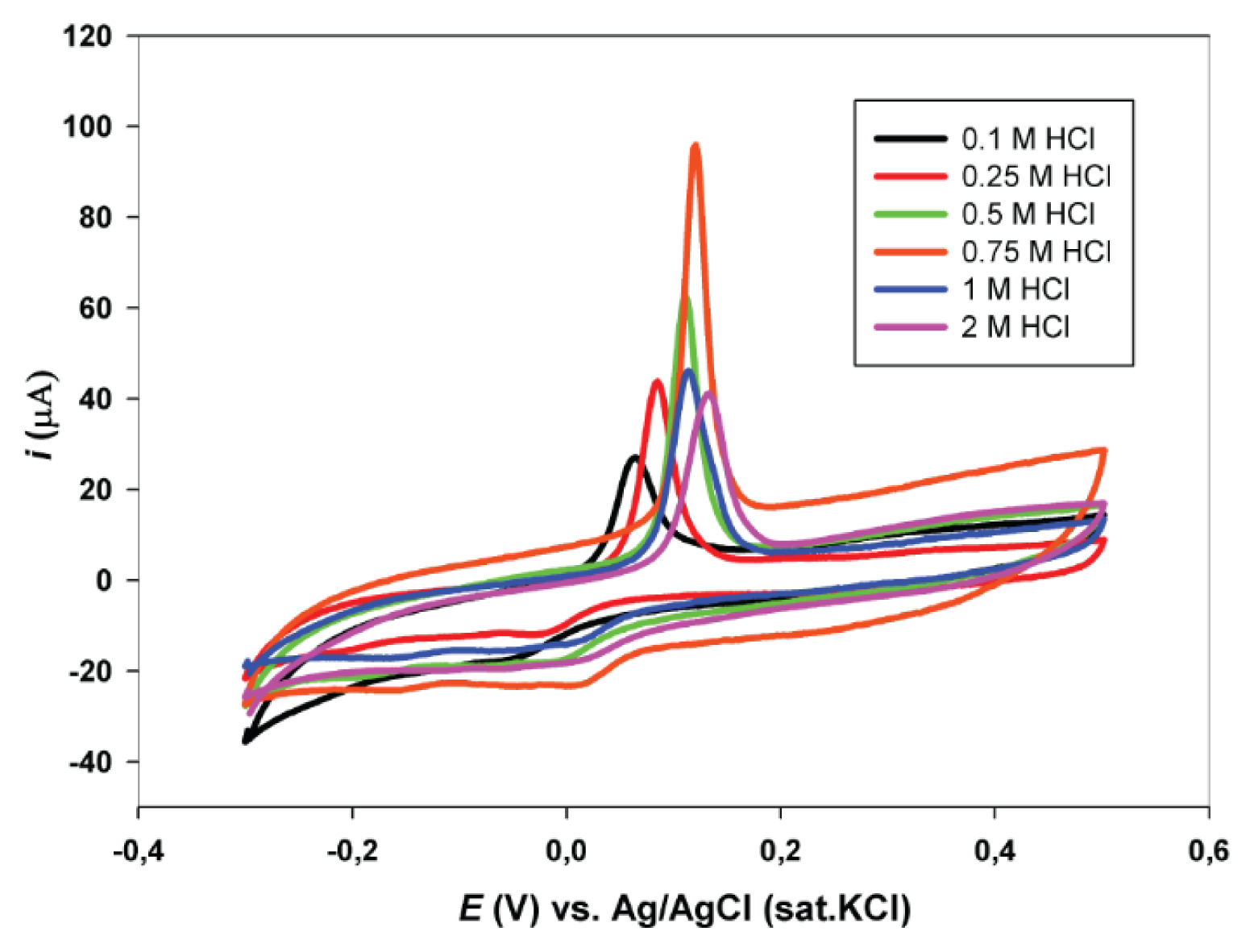

Supplement: Figure S3 — Cylic voltammograms for electooxidation of 0.1 mmol L−1 As(III) on electrode at various HCl concentrations. [file turkjchem-46-6-2123s3.tif]

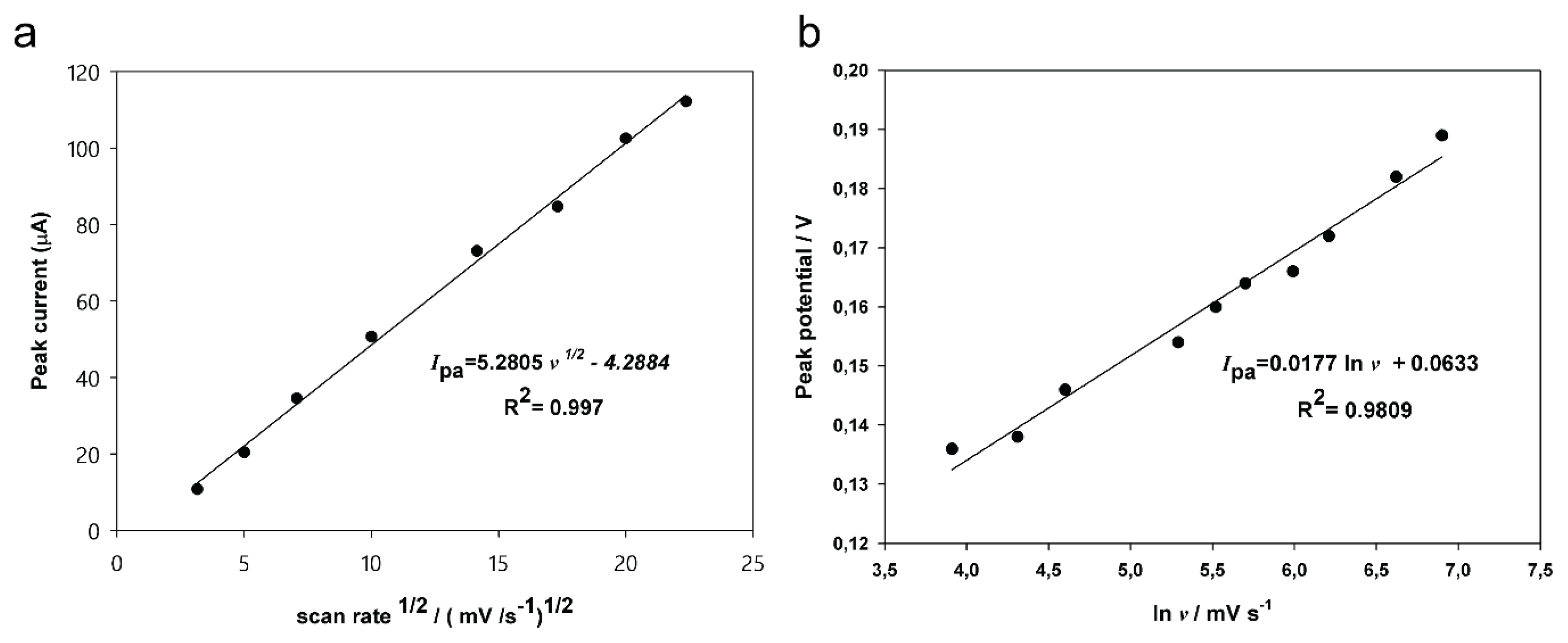

Supplement: Figure S4 — a. The plots of oxidation peak currents of As(III) versus the square root of scan rate, b. The relationship between peak potential and ln ν. [file turkjchem-46-6-2123s4.tif]

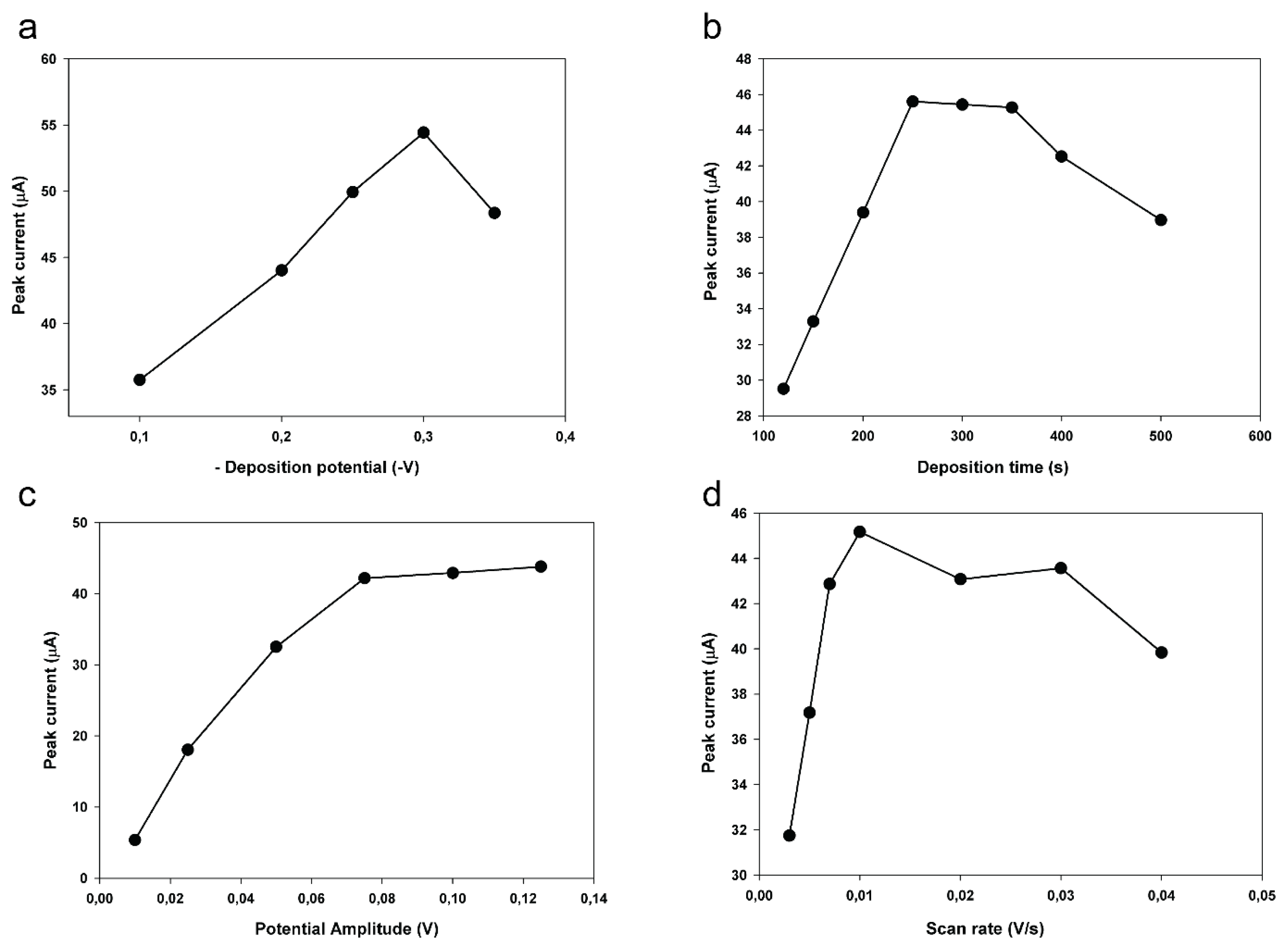

Supplement: Figure S5 — Effect of a. Deposition potential, b. deposition time, c. pulse amplitude, d. scan rate on peak currents of 5.0 μmol L−1 As(III) in 0.75 mol L−1 HCl. [file turkjchem-46-6-2123s5.tif]

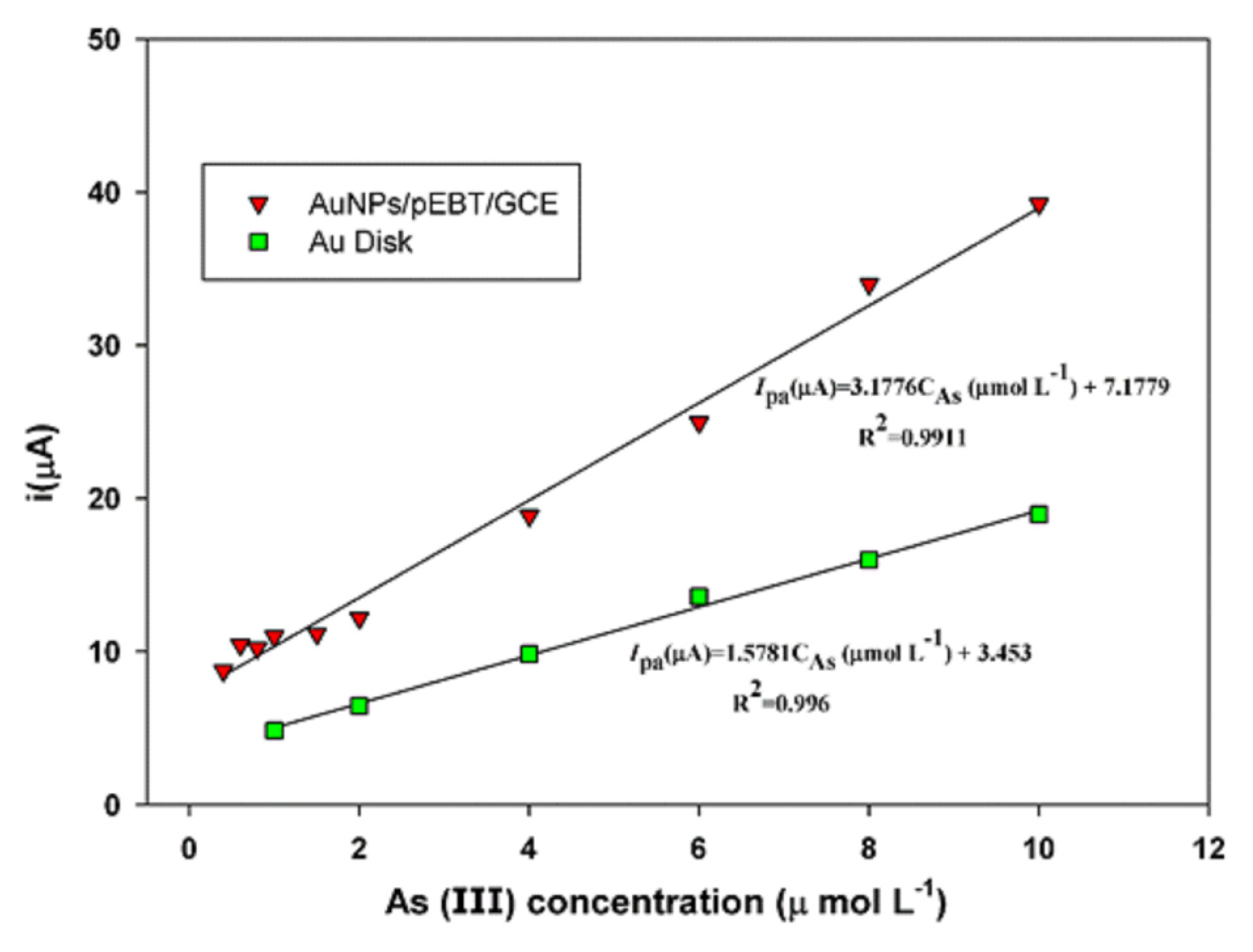

Supplement: Figure S6 — Calibration curve of As(III) for AuNPs/pEBT/GC and Au disk electrodes. [file turkjchem-46-6-2123s6.tif]
